# Bioequivalence evaluation of two cariprazine hard capsule formulations

**DOI:** 10.1007/s00210-026-05017-1

**Published:** 2026-02-14

**Authors:** Mamdouh R. Rezk, Kamal A. Badr, Ahmed Abd El Hamid Hosny, Aya M. Abdel Magid

**Affiliations:** 1 Pharmaceutical Analytical Chemistry Department, Faculty of Pharmacy, Cairo University, Cairo, Egypt; 2 Advanced Research Center (ARC), Nasr City, Cairo, Egypt; 3 R&D Department, Atco Pharmaceutical Company, Cairo, Egypt; 4 Clinical Pharmacy Department, Faculty of Pharmacy, Cairo University, Kasr El-Aini St., P.O. Box 11562 Cairo, Egypt

**Keywords:** Cariprazine, Bioequivalence, Pharmacokinetics, Schizophrenia, Safety

## Abstract

Cariprazine, a third-generation antipsychotic, is indicated for schizophrenia and bipolar I disorder. Affordable formulations are essential for improving access and adherence, particularly in resource-limited settings. We aimed to assess the bioequivalence, safety, and tolerability of a newly developed generic hard capsule Vocarzine (1.5 mg) compared to a branded reference product of cariprazine hard capsule (Reagila®) under fasting conditions in healthy adult participants. In a randomized, open-label, single-dose, two-period, two-sequence crossover design, 30 healthy male and female participants received a single oral dose of either the test or reference product, with 4-week washout period. Pharmacokinetics were evaluated using validated LC–MS/MS methods. Bioequivalence was concluded if the 90% confidence intervals (CIs) for the geometric mean ratios (GMRs) of peak plasma concentration (C_max_) and area under the concentration–time curve from time zero to 72 h (AUC_0-72h_) fell within 80–125%. Twenty-nine participants completed the study. The GMR (90% CI) for C_max_ was 91.92% (85.07–99.33%) and for AUC_0-72h_ was 92.85% (88.86–97.02%), both within the bioequivalence acceptance range (80–125%). Both formulations were well tolerated; mild nausea was the most common adverse event, and no serious adverse events were reported. The two hard capsule formulations were bioequivalent, in terms of rate and extent of absorption, with a comparable safety profile, supporting the use of vocarzine as a safe, effective, and potentially cost-saving alternative. **Clinical trial number:** The ClinicalTrials.gov registration number is NCT07121868, retrospectively registered on August 13, 2025.

## Introduction

Schizophrenia is a complex psychiatric disorder characterized by positive, negative, and cognitive symptoms, with negative symptoms being particularly resistant to treatment and closely linked to poor functional outcomes. Cariprazine, a third-generation antipsychotic, acts as a dopamine D3/D2 receptor partial agonist with preferential affinity for D3 receptors, and additional activity as a 5-HT1A partial agonist and 5-HT2B antagonist. This pharmacological activity offers potential benefits for treating negative and cognitive symptoms, along with maintaining overall symptom control (Selvan et al. 2024). Cariprazine is approved for the treatment of schizophrenia and bipolar I disorder, including acute manic or mixed episodes, bipolar depression, and adjunctive therapy for major depressive disorder (Veselinović, Paulzen and Gründer 2013; European Medicines Agency 2017; Stahl, Laredo and Morrissette 2020).

Consistent evidence from clinical trials and real-world studies has shown that cariprazine is as effective as other atypical antipsychotics for positive symptoms and offers superior benefits for negative symptoms and functional outcomes, including in patients with dual diagnoses (Németh et al. 2017; Earley et al. 2019; Rancans et al. 2021). Meta-analyses confirm cariprazine’s favorable risk–benefit profile and tolerability, supporting its widespread clinical use (Huhn et al. 2019). It is available as oral capsules in 1.5 to 6 mg strengths for once-daily dosing, with or without food, and orodispersible tablet formulations offer an alternative for patients with swallowing difficulties or specific administration preferences (Meszár et al. 2024). Due to its extended half-life and unique receptor profile, cariprazine promotes better adherence and sustained symptom control, making it a valuable option for the long-term care of schizophrenia.

Cariprazine is rapidly and well absorbed following oral administration, reaching peak plasma concentrations (C_max_) approximately 3 to 6 h post-dose. Its oral bioavailability is high, and food has no clinically relevant effects on its absorption. The drug exhibits extensive distribution, with a large apparent volume of distribution (~ 20 L/kg) and is highly bound to plasma proteins (> 98%). Cariprazine undergoes extensive hepatic metabolism, primarily via cytochrome P450 3A4 (CYP3A4), with minor involvement of CYP2D6. It is metabolized into two major active metabolites, desmethyl cariprazine (DCAR) and didesmethyl cariprazine (DDCAR), both of which contribute to the overall pharmacological effect. Due to the long half-lives of cariprazine (2–4 days) and DDCAR (1–3 weeks), steady-state concentrations are achieved slowly, and drug exposure persists for several weeks after discontinuation. At steady state, DDCAR accounts for approximately 64% of total cariprazine exposure, making it the predominant active moiety. Elimination is predominantly hepatic, with minimal renal excretion of unchanged drug (European Medicines Agency 2017; Periclou et al. 2021).

Adherence to antipsychotic treatment remains a major challenge in the long-term management of schizophrenia, with non-adherence rates estimated to reach up to 80%. Factors such as limited patient engagement in treatment decisions, unacceptable side effects, and complex regimens contribute to poor medication adherence (Byerly, Nakonezny and Lescouflair 2007; Cooper et al. 2007; Kane, Kishimoto and Correll 2013). Oral formulations such as capsules, and orodispersible tablets are generally preferred, especially early in treatment, and offer a non-invasive, familiar option that supports flexibility in addressing individual patient needs and improves medication acceptance, particularly in patients with swallowing difficulties, aversion to injections, or formulation-specific preferences (Montgomery et al. 2012; Meszár et al. 2024). The availability of a generic capsule formulation further enhances access and treatment continuity. Generic products reduce healthcare costs and allow broader access, especially in low- and middle-income countries where affordability is a major barrier (AbdelMagid, Badr and Rezk 2025; Rezk et al. 2025). Offering bioequivalent generics in multiple oral forms supports individualized care, encourages adherence, and promotes better long-term outcomes in the management of schizophrenia (Kane, Kishimoto and Correll 2013; Oldfield et al. 2025).

From a regulatory perspective, the approval of generic formulations of cariprazine requires demonstration of bioequivalence to the reference product, Reagila®, based on comparative pharmacokinetic (PK) evaluation of the parent compound. Given cariprazine’s prolonged elimination half-life and the formation of active metabolites, international regulatory agencies, including the U.S. Food and Drug Administration (FDA) and the European Medicines Agency (EMA), permit the use of truncated area under the concentration–time curve (AUC) designs for immediate-release formulations when assessing bioequivalence (Food and Drug Administration (FDA) 2021; International Council for Harmonisation of Technical Requirements for Pharmaceuticals for Human Use (ICH) 2024). The FDA product-specific guidance for cariprazine recommends bioequivalence assessment based on C_max_and truncated AUC of the parent drug, without mandatory evaluation of active metabolites, provided that linear PK are demonstrated. A recently published bioequivalence study comparing an alternative cariprazine orodispersible tablet with the reference hard capsule has confirmed the suitability of this approach in healthy participants (Meszár et al. 2024). Despite the clinical relevance of cariprazine, published bioequivalence studies of generic formulations remain limited in the literature, underscoring the need for well-conducted comparative PK studies to support regulatory approval and expand access to this therapy. This study aimed to evaluate the bioequivalence, safety, and tolerability of a newly developed generic cariprazine 1.5 mg hard gelatin capsule (Vocarzine) compared to the reference product Reagila® in healthy Egyptian participants under fasting conditions.

## Methods

### Study population

All phases of the bioequivalence study were conducted at Advanced Research Center (ARC), Cairo, Egypt, between August 2024 and January 2025. The study enrolled healthy male and female participants aged between 18 and 55 years with a body mass index (BMI) ranging from ≥ 18.5 to < 30 kg/m^2^. Participants were eligible if they were in good general health, with no clinically significant medical conditions as determined by the principal investigator, based on comprehensive evaluations including medical history, electrocardiogram (ECG), vital signs, physical and anthropometric examinations, and laboratory testing. Participants were also required to test negative for human immunodeficiency virus (HIV), hepatitis B, hepatitis C, and drug abuse. Female participants were required to agree to use effective contraception throughout the study period and for at least 30 days after completion. Participants were instructed to abstain from alcohol, grapefruit or grapefruit-containing products, and caffeine-containing beverages (including coffee) for at least 48 h prior to dosing and throughout each study period, to minimize potential PK interference. Only non-smokers or mild smokers (defined as < 5 cigarettes per day) were eligible for inclusion. Smoking was not permitted during the study period, and the participants were instructed to abstain from smoking throughout the confinement period.

The exclusion criteria included known hypersensitivity to the study drug; any clinical signs of organ dysfunction; a history of serious illnesses that could affect study outcomes; clinically significant abnormal laboratory findings; history of alcohol misuse; a positive urine drug abuse screening for amphetamines (AMP), barbiturates (BAR), benzodiazepines (BZO), cannabinoids (THC), cocaine, opiates (morphine [MOP]), or tramadol (TRA); heavy smoking; pregnancy or breastfeeding in women; consumption of grapefruit or grapefruit juice within 48 h prior to dosing or during the study period; and recent use of systemic medications within two weeks or within six elimination half-lives of the drug (whichever was longer).

### Ethical considerations

The study protocol was approved by the Independent Ethics Committee (IEC) of ARC on August 10th, 2024 (IEC No.: IEC_100824_01). The study was retrospectively registered on Clinicaltrials.gov under the identifier NCT07121868 on August 13, 2025, owing to administrative timing constraints; however, this did not affect ethical oversight, participant enrollment, or study conduct. All procedures involving human participants were conducted in accordance with the ethical standards outlined by the institutional ethics committee, the International Conference on Harmonisation’s Good Clinical Practice (ICH-GCP) guidelines (Therapeutic Goods Administration (TGA) 2018), and the Declaration of Helsinki and its subsequent amendments (2013) or equivalent ethical standards (World Medical Association 2013). Prior to the commencement of any study-related procedures, written informed consent was obtained from each participant. The study objectives, procedures, potential benefits, and risks were clearly explained to ensure full understanding before consent was obtained. All biological samples and data collected were used solely for the purposes of this study, with strict measures taken to protect the confidentiality of the participants.

### Drug products

In this study, the test formulation was Vocarzine (cariprazine hydrochloride equivalent to 1.5 mg cariprazine) in hard gelatin capsule form (batch no. 232591, expiration date: February 2026), produced by Atco Pharma for Pharmaceutical Industries, Egypt. It was compared with the reference product, Reagila® (cariprazine hydrochloride equivalent to 1.5 mg cariprazine) hard gelatin capsule (batch no. U11008A, expiration date: January 2026), manufactured by Gedeon Richter, Hungary and marketed by Recordati Pharma GmbH, Germany. The study was conducted using a 1.5 mg dose, as higher strengths were not well tolerated by healthy participants. In line with bioequivalence guidelines, lower strengths may be used when safety is a concern. Due to cariprazine’s linear PK across the 1.5–6 mg range, results at 1.5 mg are extrapolatable to higher doses as per Food and Drug Administration (FDA) draft guidance for cariprazine, provided that formulation proportionality across strengths is demonstrated (Food and Drug Administration (FDA) 2019). Prior to the clinical phase, in vitro testing was conducted for the 1.5 mg strength using the specified batches, confirming the pharmaceutical equivalence of the test product (Vocarzine 1.5 mg) and the reference product (Reagila® 1.5 mg).

### Study design

This was a single-center, open-label, randomized, single-dose, two-sequence, two-period crossover bioequivalence study conducted under fasting conditions in healthy participants. The pre-randomization phase included a screening period (from Day −30 to Day −3) and a baseline assessment on (Day −1), the day before dosing in the first treatment period. Participants who met the eligibility criteria during this phase were randomized. This phase comprised two treatment periods, separated by a four-week washout period to avoid the carryover effect, and concluded with a follow-up visit on day 32, following administration of the final study dose.

**Fig. 1:**
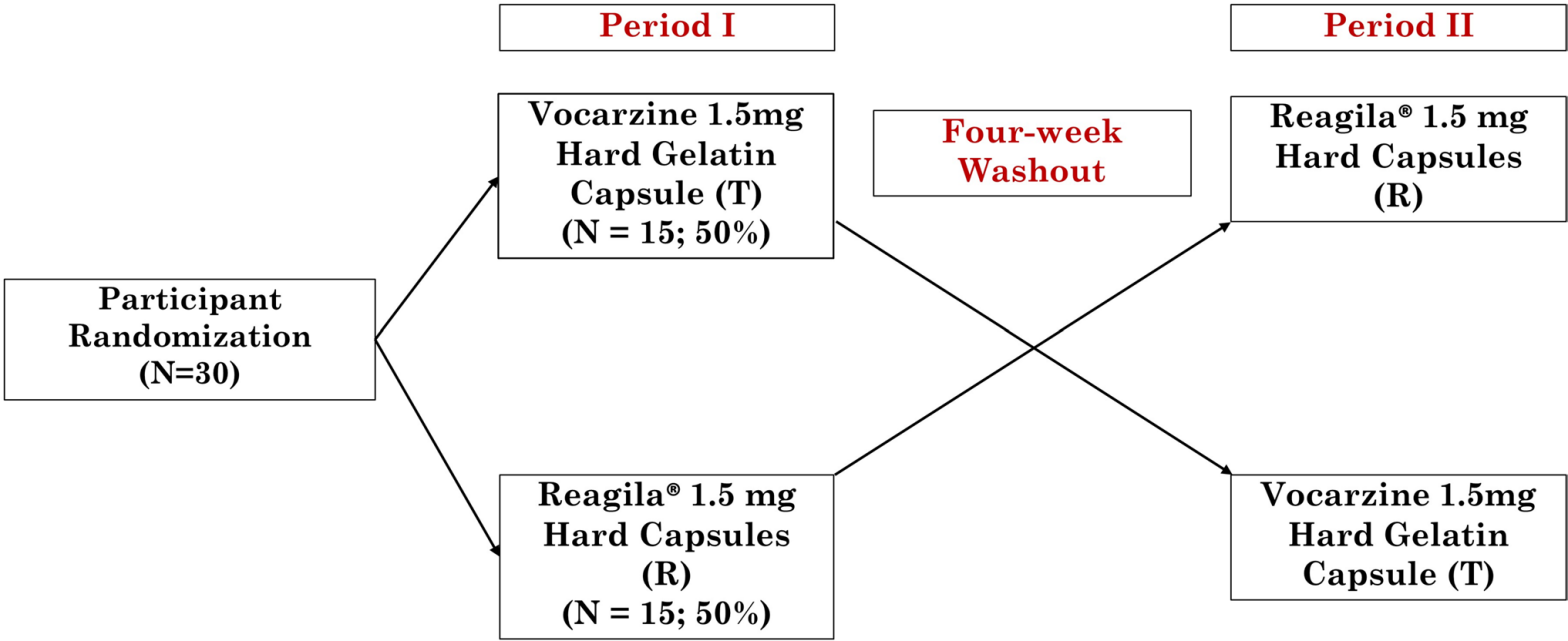
Summary of Cariprazine Hard Capsule (1.5 mg) bioequivalence study design

During each study period, participants were randomly assigned in a 1:1 ratio to receive a single capsule of either the test or reference product under fasting conditions, following a two-period, two-sequence crossover design. Participants received either the test product in the first period followed by the reference product in the second period (TR), or vice versa (RT) (Fig. 1). The randomization schedule was developed by an independent statistician based on the planned sample size outlined in the study protocol, considering the number of treatments and crossover design. Randomization was performed using R statistical software (version 4.2.1) using a computer-generated random sequence. The allocation sequence was generated using a fixed random seed (Seed Number: 5487698) to ensure reproducibility.

Participants fasted overnight for a minimum of 10 h prior to receiving a single oral dose of cariprazine. On the morning of each study period, fluid intake was restricted for one hour before dosing. A single 1.5 mg cariprazine capsule (test or reference formulation) was administered orally with 240 mL of water. Fluid intake was permitted again one hour after dosing. To maintain uniform conditions across the treatment periods, all participants received a standardized diet, with meals and beverages served at consistent times during each period. The first and second meals were provided at 4- and 8-h post-dose, respectively. The investigational products were administered at the trial site under the supervision of the study team, followed by checks of the oral cavity and hands for compliance. Participants remained under in-house supervision for 12 h post-dose during each study period, after which they were discharged from the study site and returned for scheduled terminal PK sampling and follow-up assessments.

### Pharmacokinetic analysis

In each study period, a total of 22 blood samples (5 mL each) were collected into EDTA-treated tubes according to the following schedule: pre-dose (*t* = 0), and at 0.5, 1, 2, 2.5, 3, 3.5, 4, 4.5, 5, 5.5, 6, 6.5, 7, 7.5, 8, and 10, 12 (Day 1), 24, 36 (Day 2), 48 (Day 3), and 72 h (Day 4) post-dose. Any deviations from the scheduled sampling times were documented precisely, and PK calculations were based on the actual sampling times. Blood samples were collected up to 12 h post-dose via a cannula inserted into a forearm vein, whereas samples at 24, 36, 48, and 72 h were obtained through venipuncture. Immediately after collection, the samples were centrifuged for 5 min at 3500 rpm and 4 °C using a PRO-analytical centrifuge (Centurion Scientific Limited-PRO-Analytical CR(International 2000), PRO Analytical Cooling, UK), with a maximum allowable interval of ≤ 30 min between collection and centrifugation to maintain sample integrity. The resulting plasma was transferred into labeled plastic tubes and stored at − 80 °C at the trial site until further analysis.

Non-compartmental PK analysis was conducted using Phoenix WinNonlin version 8.3.4 (Certara, NJ, USA) to determine the primary and secondary PK parameters. The primary PK parameters included C_max_ and AUC from time zero to 72 h (AUC_0-72h_). Due to cariprazine’s prolonged half-life, the AUC was truncated at 72 h, and AUC_0-72h_ was used in place of AUC_0-∞_for analysis as per the Guideline on the Investigation of Bioequivalence, which recommends the use of a truncated AUC for immediate-release formulations of long half-life drugs (International Council for Harmonisation of Technical Requirements for Pharmaceuticals for Human Use (ICH) 2024), as prespecified in the study protocol. Secondary PK parameters included the time to reach maximum concentration (t_max_), terminal elimination half-life (t_₁/₂_), and last quantifiable plasma concentration (C_last_). The elimination rate constant (k_el_) was derived from the slope of the log-transformed plasma concentration versus time curve, and the half-life was calculated using the formula t_₁/₂_ = ln (2)/k_el_.

### Bioequivalence evaluation

The primary objective of this study was to evaluate the bioequivalence between the test and reference products. The bioequivalence study was designed and conducted in accordance with the requirements of the Egyptian Drug Authority (EDA) and FDA bioequivalence guidance, with consideration of the principles outlined in the ICH M13A guideline for immediate-release oral dosage forms (Egyptian Drug Authority (EDA) 2021; Food and Drug Administration (FDA) 2021; International Council for Harmonisation of Technical Requirements for Pharmaceuticals for Human Use (ICH) 2024). In accordance with the guidelines, the key PK parameters used to determine dose comparability were systemic exposure metrics, specifically AUC_0-72h_ and C_max _(Food and Drug Administration (FDA) 2021). The AUC was calculated using the linear trapezoidal method by applying linear interpolation between concentration–time data points up to the last measurable concentration (AUC_0-t_), with extrapolation of the terminal elimination phase to infinity (AUC_0-∞_) based on the terminal k_el_. The t_max_ was summarized using the median and range. For all other PK parameters, both the arithmetic mean and geometric mean (following log-transformation) were reported. To compare the test and reference products, the geometric mean ratio (GMR%) and corresponding 90% confidence intervals (CIs) were calculated for AUC_0-72h_ and C_max_. The two formulations were considered bioequivalent if the 90% CI for the GMR% of AUC_0-72h_ and C_max_fell within the accepted bioequivalence range of 80% to 125% (Food and Drug Administration (FDA) 2021).

### Bioanalytical method

Bioanalysis was conducted at ARC, Cairo, Egypt, using liquid chromatography coupled with tandem mass spectrometry (LC–MS/MS), specifically a Waters Acquity UPLC H-Class-Xevo TQD system (MA, USA) and a Waters Quattro Premier XE triple quadrupole mass spectrometer. Cariprazine concentrations in human plasma were measured using a fully validated bioanalytical method.

Chromatographic separation was performed using a Phenomenex Kinetex® EVO C_18_ column (5 µm, 4.6 × 100 mm) maintained at 45 °C, with a flow rate of 0.7 mL/min. The mobile phase consisted of 10 mM ammonium formate with 0.1% formic acid and acetonitrile in a 40:60 (v/v) ratio. Cariprazine-d6 (Cayman Chemical, Ann Arbor, MI, USA), a deuterated analog, was used as the internal standard.

The method demonstrated linear calibration curves over a concentration range of 0.05 to 5 ng/mL for cariprazine. The method was validated in accordance with the FDA’s bioanalytical method validation guidance, with alignment to the principles and requirements of the ICH M10 guideline, ensuring compliance with parameters including selectivity, precision, accuracy, linearity, calibration curve performance, stability, and carry-over (Zimmer 2014; Food and Drug Administration (FDA) 2018; International Council for Harmonisation of Technical Requirements for Pharmaceuticals for Human Use 2022). The bioanalytical personnel responsible for sample analysis were blinded to the treatment allocation and sequence to minimize analytical bias.

### Safety evaluation

Throughout the entire study, the participants were closely monitored to ensure drug safety. The principal investigator regularly inquired, on an hourly basis, whether any adverse events had occurred, and the participants were instructed to immediately report any unfavorable symptoms experienced during the study. Vital signs, including blood pressure, heart rate, respiratory rate, and body temperature, were recorded at baseline (pre-dose) and at 3, 6, 12, and 72 h after dosing. At the end of the study, a follow-up complete blood count was performed for all enrolled participants.

If any adverse events were reported, the principal investigator evaluated each case to determine its seriousness, severity and potential relationship with the investigational drug. Adverse event severity was graded according to the Common Terminology Criteria for Adverse Events (CTCAE), version 5.0, and classified as grade 1 (mild), grade 2 (moderate), grade 3 (severe), grade 4 (life-threatening), or grade 5 (death) (National Cancer Institute 2017). Given that the study medication may moderately affect the ability to drive or operate machinery, potentially causing dizziness or drowsiness, participants’ alertness levels were assessed by clinical evaluation, including assessment of orientation, responsiveness, and level of consciousness, before they were permitted to leave the study site during each treatment period (European Medicines Agency 2017).

### Sample size

No published data are available regarding the within-subject variability coefficient (CV_ws_) of cariprazine to support a formal a priori power or sample size calculation. In accordance with the Egyptian Drug Authority regulations, which require a minimum of 24 participants (Egyptian Drug Authority (EDA) 2021), the planned sample size for this study was set at 30 healthy male and female participants to allow for potential dropouts. The study was prospectively designed as a two-stage adaptive bioequivalence study. If bioequivalence was not demonstrated and/or the achieved statistical power was below 80%, an add-on phase was specified. The number of additional participants will be calculated based on the observed CV_ws_ in the completed study, assuming a test/reference (T/R) ratio of 0.95 and a significance level (α) of 0.0294.

### Statistical analysis

Statistical analyses were conducted using R software (version 4.2.1, R Foundation for Statistical Computing, Vienna, Austria). The bioequivalence assessment employed two one-sided *t*-tests (TOST), with 90% CI calculated for the log-transformed PK parameters of cariprazine to compare the test and reference formulations. For t_max_ comparisons between the two products, the nonparametric Wilcoxon signed-rank test was applied, with a significance threshold of *p* < 0.05. To evaluate the sequence, treatment, and period effects, an Analysis of Variance (ANOVA) test was conducted on the primary PK parameters, AUC_0-72h_ and C_max_ and their log-transformed values to examine potential variations related to the sequence of administration, treatment received, and specific study period. A post hoc power evaluation was performed based on the observed CV_ws_ obtained from the final dataset.

## Results

### Study population

**Fig. 2:**
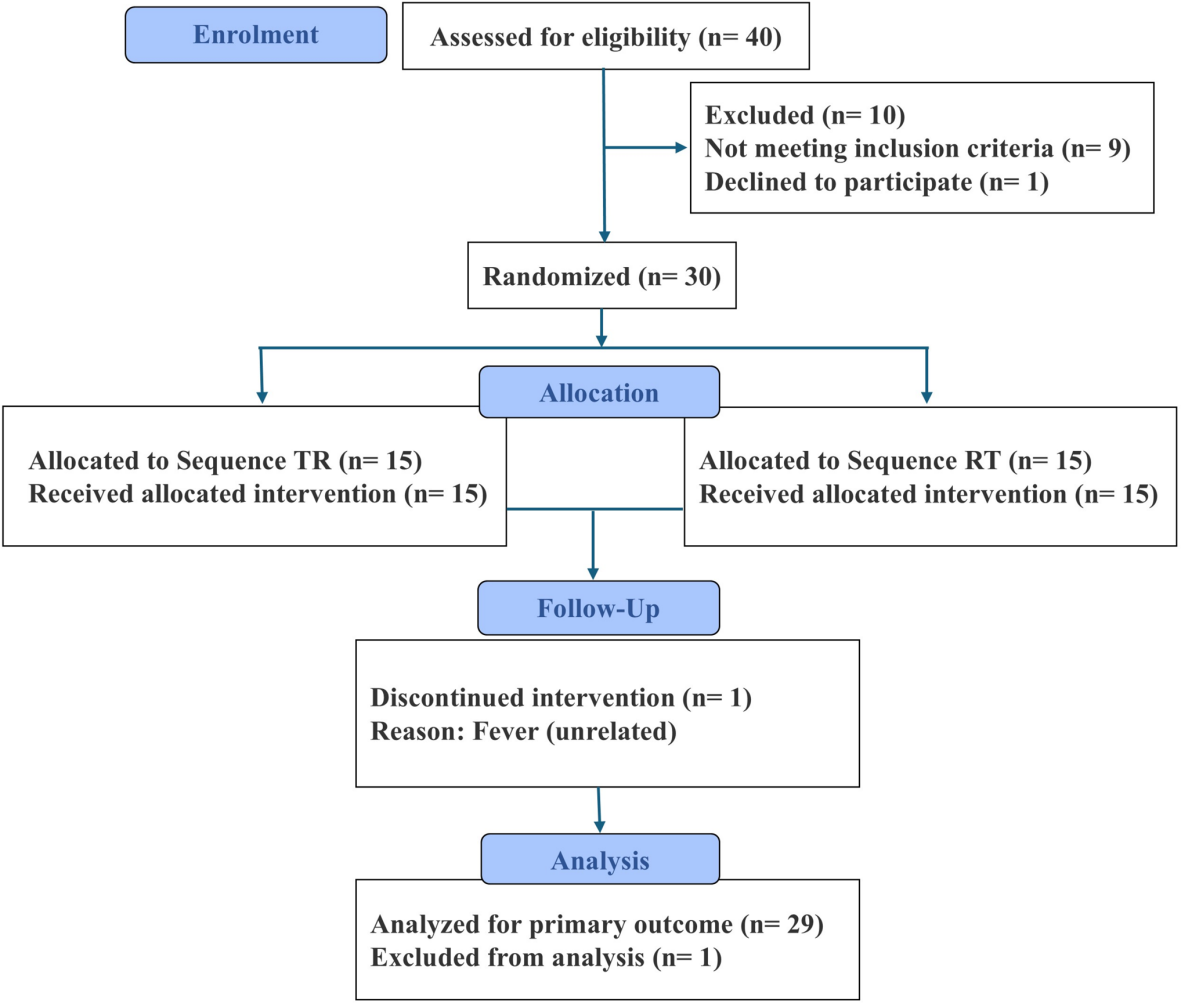
CONSORT flow diagram

 A total of 40 participants were screened for the study, of whom 10 did not meet the eligibility criteria due to reasons such as anthropometric measurements outside the acceptable protocol-defined range, positive drug tests, refusal to sign informed consent, and poor compliance. The remaining 30 participants were successfully enrolled and randomized. Of these, 29 participants completed the study, while one participant was discontinued by the principal investigator during the first treatment period because of the onset of fever (Fig. 2). The enrolled participants included both males and females (22 males and 8 females who were neither pregnant nor lactating). The mean age was 31.44 ± 10.72 years, with an average height of 168.4 ± 7.76 cm, mean body weight of 69.03 ± 9.84 kg, and mean BMI of 24.44 ± 3.74 kg/m^2^. The demographic data and baseline characteristics of the study population are presented in Table 1.

**Table 1:** Summary of demographics and baseline laboratory parameters

Parameter (unit)	Value	Parameter (unit)	Value
Age (years)	31.44 ± 10.62	Blood Urea (mg/dL)	24.14 ± 6.48
Height (cm)	168.40 ± 7.76	Creatinine (mg/dL)	0.79 ± 0.16
Weight (kg)	69.03 ± 9.84	Sodium (mEq/L)	139.45 ± 2.25
BMI (kg/m^2^)	24.44 ± 3.74	Potassium (mEq/L)	4.73 ± 0.38
Hb (g/dl)	13.77 ± 1.55	Total Bilirubin (mg/dL)	0.58 ± 0.19
RBCs (10^6^/mm^3^)	5.18 ± 0.68	ALP (U/L)	78.48 ± 18.42
Hct (%)	41.28 ± 4.87	Total Protein (g/dL)	8.08 ± 1.56
Platelet Count (10^3^/mm^3^)	275.59 ± 84.19	Cholesterol (mg/dL)	171.48 ± 54.32
WBCs (10^3^/mm^3^)	6.91 ± 2.16	Triglycerides (mg/dL)	160.52 ± 98.01
Random Blood Glucose (mg/dL)	92.10 ± 7.75	HDL-Cholesterol (mg/dL)	44.31 ± 11.4
ALT (U/L)	19.03 ± 10.4	LDL-Cholesterol (mg/dL)	95.1 ± 46.42
AST (U/L)	19.79 ± 4.70		

Data is presented as mean ± SD for 30 participants (n = 30)

Abbreviations: *ALT*, Alanine Aminotransferase; *ALP*, Alkaline Phosphatase; *AST*, Aspartate Aminotransferase; *BMI*, Body Mass Index;* Hb*, Hemoglobin; *Hc*, Hematocrit; *HDL*, High Density Lipoprotein; *LDL*, Low Density Lipoprotein; *RBCs*, Red Blood Cells; *WBCs*, White Blood Cells

### Pharmacokinetics

**Fig. 3:**
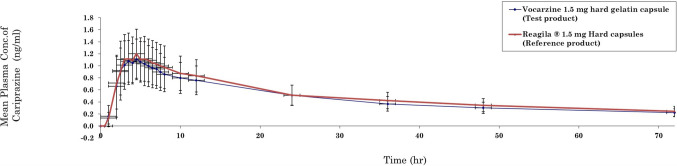
The mean plasma concentration–time profile of Cariprazine 1.5 mg hard capsule following oral administration of one capsule of both Vocarzine (Test) and Reagila® (Reference) to healthy participants under fasting conditions (N = 29)

The PK analysis included 29 participants who completed both treatment periods and had adequate plasma cariprazine concentration data for evaluation. The mean plasma concentration–time curves for cariprazine, following the oral administration of a single 1.5 mg hard gelatin capsule of Vocarzine (Test) and Reagila® (Reference), under fasting conditions, in 29 healthy Egyptian participants are illustrated in (Fig. 3). Following administration of the test and reference formulations to 29 healthy participants, the arithmetic mean C_max_ of cariprazine was 1.27 ± 0.42 ng/mL for the test product and 1.37 ± 0.39 ng/mL for the reference product. The arithmetic mean AUC_0-72h_ was 33.19 ± 9.87 ng.h/mL and 35.94 ± 10.94 ng.h/mL for the test and reference formulations, respectively. The median t_max_ was 4 h for both products, with a range of 2 to 7 h. A detailed summary of the primary and secondary PK parameters for both formulations is presented in Table 2.

**Table 2:** Summary of pharmacokinetic parameters of Cariprazine following administration of a single oral dose of Vocarzine (Test) and Reagila® (Reference) hard capsule Formukation in Egyptian healthy participants

PK Parameter, unit	Test	Reference
C_max_, ng/mL	1.27 ± 0.42	1.37 ± 0.39
C_last_, ng/mL	0.22 ± 0.07	0.24 ± 0.08
t_max_, h	4 (2–7)	4 (2–7)
AUC_0-72h_, ng.h/mL	33.19 ± 9.87	35.94 ± 10.94
AUC_0-∞,_ ng.h/mL	45.47 ± 12.98	51.06 ± 16.78
t_1/2_, h	38.81 ± 8.74	42.31 ± 12.78
K_e,_ h^−1^	0.019 ± 0.004	0.018 ± 0.005

Data are presented as arithmetic mean ± SD, except for t_max_ which are median (minimum–maximum) for 29 participants (*n* = 29)

Statistical comparison between treatments for t_max_ was performed using the Wilcoxon signed-rank test; *p* = 0.0717

*C*_*max*_, the maximum plasma concentration; *C*_*last*_, the last quantifiable plasma concentration; *t*_*max*_, the time to reach maximum plasma concentration; *AUC*_*0-72h*_, the area under the plasma concentration–time curve up from time zero to the 72 h; *AUC*_*0-∞*,_ the area under the plasma concentration–time curve from time zero up to infinity; t_1/2_, the elimination half-life; K_e_, elimination rate constant; *PK*, Pharmacokinetic

### Bioequivalence and statistical analysis

The results of the bioequivalence assessment of the two formulations are presented in Table 3. The study was conducted according to a predefined two-stage adaptive bioequivalence design. Following completion of the first stage, the 90% CI for the GMRs of C_max_ and AUC_0-72h_ were fully contained within the accepted bioequivalence range of 80–125%. The within-subject variability coefficient of variation (CV_ws_) for cariprazine was 9.83% for AUC_0-72h_ and 17.44% for C_max,_ corresponding to an achieved study power of approximately 90%. As the bioequivalence criteria were satisfied at the first stage and the study was sufficiently powered, continuation to the second stage was not required, and the study was completed after the first stage.

**Table 3:** Bioequivalence evaluation of cariprazine after single oral dose administration of one capsule of test product (T) Vocarzine 1.5 mg hard capsule versus one capsule of reference product (R) Reagila® 1.5 mg hard capsule under fasting conditions

PK Parameter (Unit)	Geometric Means		GMR% Test/Reference	90% CI	CV_ws_
	Test Vocarzine	Reference Reagila®			
C_max_ (ng/mL)	1.21	1.33	91.92	85.07–99.33	17.44%
AUC_0-72h_ (ng.h/mL)	31.74	34.38	92.85	88.86–97.02	9.83%

N = 29 participants

Abbreviation: *AUC*_*0-72h*_, Area under the plasma concentration–time curve from time zero to 72 h; *CI*, Confidence interval; *C*_*max*_, Maximum observed plasma concentration; *CV*_*ws*_, Within-subject coefficient of variation; *GMR*, Geometric mean ratio

The GMR% of the test to reference formulations for cariprazine, along with the corresponding 90% CI was 91.92% (85.07–99.33%) for C_max_ and 92.85% (88.86–97.02%) for AUC_0-72h._ Both CI values fell within the predefined bioequivalence acceptance range of 80–125%. It has been shown that there was no statistically significant sequence effect for logarithmically Ln-transformed C_max_ or AUC_0-72h_ (all at *p* > 0.05, ANOVA). A statistically significant period effect was observed for both Ln-C_max_ and Ln-AUC_0-72h_ (*p* < 0.05). The treatment effect was not statistically significant for Ln-C_max_ (*p* > 0.05), whereas a statistically significant formulation effect was observed for Ln-AUC_0-72h_ (*p* < 0.05). Importantly, bioequivalence was concluded based on the TOST procedure, with the 90% CI for the GMRs of C_max_ and AUC_0-72h_ fully contained within the predefined acceptance range of 80–125%. Using a nonparametric analysis (Wilcoxon signed-rank test), no statistically significant difference was found between the test and reference formulations in terms of t_max_, with a *p*-value of 0.0717.

### Safety evaluation

Throughout both study periods, almost all reported adverse events were mild, drug-related, and no serious adverse events occurred. A summary of all adverse events observed during the study is presented in Table 4. In the first period, five out of thirty participants (16.67%) reported nausea as an adverse event, while in the second period, nausea was reported by six participants (20%). During the follow-up phase, complete blood count (CBC) results revealed mild laboratory-related adverse events in some participants: low hemoglobin levels in eight participants (26.67%), reduced red blood cell count in four participants (13.33%), thrombocytopenia in one participant (3.33%), and leucopenia in one participant (3.33%). In addition, one participant (3.33%) developed moderate fever during the first treatment period after receiving the investigational product; the event was assessed as unrelated to the study drug, the participant received appropriate medical management and was subsequently discontinued from the study by the principal investigator. All vital signs, including blood pressure, heart rate, respiratory rate, and body temperature, remained within normal limits during both study periods.

**Table 4:** Summary of adverse events and laboratory findings

Type of event	Formulation	Number of participants (frequency (%))	Severity
Summary			
No. of patients with grade 3–4 AE	-	None	-
No. of patients with a SAE	-	None	-
Death	-	None	-
Clinical adverse events			
Nausea	Test	5 (16.67%)	Mild
Nausea	Reference	6 (20%)	Mild
Fever	Reference	1 (3.33%)	Moderate
Follow-up laboratory findings			
Low Hb	Both (follow-up)	8 (26.67%)	Mild
Reduced RBCs count	Both (follow-up)	4 (13.33%)	Mild
Thrombocytopenia	Both (follow-up)	1 (3.33%)	Mild
Leukopenia	Both (follow-up)	1 (3.33%)	Mild

N = 30 participants

Abbreviations: *AE*, Adverse event; *Hb*, Hemoglobin; *RBCs*, Red Blood Cells; *SAE*, Serious Adverse Event

## Discussion

This study demonstrated that the newly developed generic hard capsule formulation of cariprazine (vocarzine) is bioequivalent to the reference product Reagila® as reflected by the C_max_ and AUC_0-72h_values. The 90% CI for the GMR of these PK parameters fell within the predefined bioequivalence acceptance range of 80–125% in accordance with international regulatory guidelines (Food and Drug Administration (FDA) 2021; International Council for Harmonisation of Technical Requirements for Pharmaceuticals for Human Use (ICH) 2024). The observed GMR values for C_max_ and AUC_0-72h_were approximately 92%, which, although slightly below unity, remained well within the regulatory acceptance limits and were commonly observed in bioequivalence studies where minor formulation- or process-related differences existed. Importantly, such deviations are not interpreted as clinically relevant when CIs fully meet bioequivalence criteria, as regulatory decisions are based on interval inclusion rather than proximity to 100% (Egyptian Drug Authority (EDA) 2021; Food and Drug Administration (FDA) 2021; International Council for Harmonisation of Technical Requirements for Pharmaceuticals for Human Use (ICH) 2024). The within-subject variability was acceptably low, supporting the robustness of the crossover design and the consistency of cariprazine exposure between formulations. Additionally, the generic formulation is equally safe and well tolerated as the original product with similar risk profile, consistent with the cariprazine summary of product characteristics and typical for the class of atypical antipsychotics (European Medicines Agency 2017).

Cariprazine’s complex PK profile, including its long half-life and the presence of active metabolites, typically presents challenges in designing bioequivalence studies. However, current regulatory guidance allows for truncated AUC designs when evaluating drugs with extended half-lives in immediate-release formulations (Egyptian Drug Authority (EDA) 2021; International Council for Harmonisation of Technical Requirements for Pharmaceuticals for Human Use (ICH) 2024). The choice of a 72-h sampling window in this study was prespecified in the study protocol and directly aligned with international bioequivalence guidelines, rather than being arbitrary. It was therefore appropriate and sufficient to capture meaningful exposure comparisons, especially considering cariprazine’s linear PK across therapeutic doses (Periclou et al. 2021).

The study enrolled healthy participants, a common practice in bioequivalence research on antipsychotics, as it helps reduce inter-individual variability caused by underlying disease, differences in metabolism, or concurrent medications (Food and Drug Administration (FDA) 2021). While bioequivalence studies in patients with schizophrenia are uncommon due to ethical and logistical limitations, population PK analyses have shown that cariprazine follows a three-compartment model with zero-order input to a depot compartment and first-order absorption and elimination, while its active metabolites DCAR and DDCAR follow two-compartment models with linear elimination, with similar exposure profiles observed in both healthy participants and patients regardless of covariates such as weight, sex, renal function, or CYP2D6 metabolizer status (Periclou et al. 2021). Bioequivalence assessment in this study was based on PK evaluation of the parent compound, cariprazine, without direct measurement of its active metabolites (DCAR and DDCAR). This approach is consistent with regulatory standards, including the FDA product-specific guidance for cariprazine, which does not require metabolite assessment for bioequivalence determination. Although DCAR and DDCAR have long elimination half-lives, their formation is predictable, dose-proportional, and directly dependent on systemic exposure to the parent drug; therefore, comparable cariprazine exposure is expected to result in comparable metabolite exposure. Consequently, omission of metabolite analysis is not expected to affect the validity of the bioequivalence conclusions (Food and Drug Administration (FDA) 2019; Periclou et al. 2021).

Both formulations were generally well tolerated, with no serious adverse events observed and no clinically meaningful differences in adverse event frequency or severity were noted between the test and reference formulations. Adverse events were systematically graded using CTCAE version 5.0 and quantitatively summarized by treatment and study period (National Cancer Institute 2017). Mild nausea was the most frequently reported side effect, aligning with the established safety profile of cariprazine, which commonly includes gastrointestinal disturbances, drowsiness, and extrapyramidal symptoms (European Medicines Agency 2017). Regarding laboratory assessment, end-of-study hematological assessment was included as part of routine post-study evaluation in accordance with the requirements of the Institutional Review Board and the Egyptian regulatory authority for bioequivalence studies conducted in healthy participants. This approach is commonly adopted to ensure the absence of delayed laboratory changes, particularly given the prolonged elimination half-life of cariprazine and its active metabolites, which may persist for several weeks after dosing. In the present study, follow-up complete blood count assessments revealed only mild and transient laboratory changes without clinical relevance, and no hematological safety signals or serious adverse events were identified. These findings are consistent with the well-established favorable safety profile of cariprazine reported in previous clinical studies in schizophrenia and bipolar disorder (Veselinović, Paulzen and Gründer 2013; Stahl, Laredo and Morrissette 2020; Rancans et al. 2021).

Given the long elimination half-life of cariprazine and its active metabolites, the adequacy of the wash-out period warrants consideration (European Medicines Agency 2017; Periclou et al. 2021). In the present study, pre-dose plasma samples collected at the start of the second treatment period showed either undetectable or negligible cariprazine concentrations, indicating minimal residual exposure and supporting the sufficiency of the wash-out interval. Furthermore, the frequency and pattern of adverse events were comparable between the first and second study periods, with no increase in either the incidence or severity of adverse events in the second period. These findings suggest the absence of clinically relevant carryover effects and are consistent with previous PK and safety data despite the prolonged terminal half-lives of cariprazine metabolites (Periclou et al. 2021).

The availability of a bioequivalent generic formulation like vocarzine presents notable clinical and economic benefits. Although this study did not include a formal pharmacoeconomic evaluation, the introduction of a generic cariprazine formulation may represent a potentially cost-saving alternative to branded therapy, particularly in resource-limited settings where access to novel antipsychotic agents can be constrained (Oldfield et al. 2025). In psychiatric care, providing multiple oral bioequivalent options is essential, given the persistent challenges with medication adherence driven by factors such as side effects, pill burden, and individual preferences (Byerly, Nakonezny and Lescouflair 2007; Cooper et al. 2007; Kane, Kishimoto and Correll 2013). Due to its strong D3 receptor affinity and prolonged receptor occupancy, cariprazine is particularly effective for treating predominant negative symptoms of schizophrenia, an area often under-treated by other antipsychotics (Németh et al. 2017; Earley et al. 2019; Selvan et al. 2024). Improved availability through generic formulations may therefore facilitate broader access to an effective treatment option, while maintaining therapeutic equivalence and safety.

Nevertheless, several limitations should be acknowledged. This bioequivalence study was conducted in healthy participants using a single-dose design, which may not fully capture steady-state PK or tolerability in patients with schizophrenia or bipolar disorder. In addition, owing to the long half-life of cariprazine and its active metabolites, systemic exposure was assessed using a truncated AUC_0-72h_ rather than complete AUC_0-∞_, in line with international regulatory guidance for long half-life drugs. Finally, the moderate sample size and short duration of follow-up limit the ability to detect rare or long-term adverse events. Despite these limitations, the study design and analytical approach were appropriate for the primary objective of demonstrating bioequivalence between the two formulations.

## Conclusion

The two 1.5 mg cariprazine hard capsule formulations, Vocarzine and Reagila®, demonstrated bioequivalence in terms of C_max_ and AUC_0-72h_, within the accepted bioequivalence range of 0.8 to 1.25. Both formulations were found to be safe and well tolerated. The approval of the generic product offers a potentially cost-effective alternative for patients in need of a non-oral dosage form.

## Data Availability

All data supporting the results reported in the manuscript are available upon request from the authors.
